# Differential responses to avian pathogenic *E. coli* and the regulatory role of splenic miRNAs in APEC infection in Silkie chickens

**DOI:** 10.3389/fcimb.2024.1358216

**Published:** 2024-03-12

**Authors:** Wenqing Li, Wanli Li, Pinhui Wu, Wei Jin, Lin Yuan, Bingxun Wang, Shengli Li, Xiangtao Kang

**Affiliations:** ^1^ College of Life Science, Henan Agricultural University, Zhengzhou, China; ^2^ The Shennong Laboratory, Henan Academy of Agricultural Sciences, Zhengzhou, China; ^3^ Institute of Animal Science, Henan Academy of Agricultural Sciences, Zhengzhou, China; ^4^ Henan Key Laboratory of Farm Animal Breeding and Nutritional Regulation, Henan Academy of Agricultural Sciences, Zhengzhou, China; ^5^ College of Animal Science and Technology, Henan Agricultural University, Zhengzhou, China; ^6^ Henan Key Laboratory for Innovation and Utilization of Chicken Germplasm Resources, Henan Agricultural University, Zhengzhou, China

**Keywords:** avian pathogenic *Escherichia coli*, Silkie chickens, median lethal dose, miRNAs, infection

## Abstract

Avian pathogenic *Escherichia coli* (APEC) is a bacterial disease that harms the poultry industry worldwide, but its effect on Chinese Silkie has not been reported. Studies on whether there are differences in Silkie individual resistance to APEC and the regulatory role of spleen miRNAs lay the foundation for strategies against APEC. Therefore, 270 Silkie chickens were infected with the median lethal dose of an *E. coli* O1, O2, and O78 mixture. These chickens were divided into a susceptible group (Group S) and a recovery group (Group R) according to whether they survived 15 days postinfection (dpi). Moreover, 90 uninfected APEC Silkie served as controls (Group C). The splenic miRNA expression profile was examined to evaluate the role of miRNAs in the APEC infection response. Of the 270 Silkies infected with APEC, 144 were alive at 15 dpi. Cluster analysis and principal component analysis (PCA) of splenic miRNAs revealed that the four Group R replicates were clustered with the three Group C replicates and were far from the three Group S replicates. Differentially expressed (DE) miRNAs, especially gga-miR-146b-5p, play essential roles in immune and inflammatory responses to APEC. Functional enrichment analyses of DEmiRNAs suggested that suppression of immune system processes (biological processes) might contribute to susceptibility to APEC and that FoxO signaling pathways might be closely associated with the APEC infection response and postinfection repair. This study paves the way for screening anti-APEC Silkies and provides novel insights into the regulatory role of miRNAs in APEC infection.

## Introduction

Xichuan Silkie chickens are a special local breed found in Xichuan County, Henan Province, China. These chickens are distinguished by white feathers, a black mouth, black legs, black skin, black bone, black meat, and a green eggshell. This breed has been recorded in the Annals of Poultry and Livestock Genetic Resources of China. From a production standpoint, Silkies represent an essential part of the bird industry in China because they are highly valued as a source of medicine, food, and eggs (Higginbottom et al., 2018; Liu et al., 2022). In recent years, the development of local breeds has focused mainly on improving their production performance, but some local farmers have reported occasional outbreaks of the avian pathogenic *Escherichia coli* (APEC) in black-bone chickens.

APEC, a potential foodborne zoonotic pathogen, commonly infects poultry and causes colibacillosis (Sun et al., 2022b). This disease is one of the leading causes of mortality and morbidity in poultry (Guabiraba and Schouler, 2015; Kathayat et al., 2021). APEC infection leads to diverse local and systemic infections in poultry, either as a primary pathogen or as a secondary pathogen in birds, with predisposing factors (Kathayat et al., 2021; Sun et al., 2022b). Among the multiple APEC serotypes, three (O1, O2, and O78) account for more than 80% of all APEC infection outbreaks (Dho-Moulin and Fairbrother, 1999; Ghunaim et al., 2014). The outbreak of APEC in China in recent years has also been dominated by these three serotypes (Yan et al., 2023). Furthermore, APEC can enter the food supply through contaminated meat and eggs, resulting in human exposure (Sandford et al., 2011; Kathayat et al., 2021). APEC has been estimated to cost the global poultry industry hundreds of millions of dollars in economic losses (Ghunaim et al., 2014). In the prevention and control of avian colibacillosis caused by APEC, vaccines and antibiotics are traditionally used as practical measures. However, the emergence of bacterial resistance under the pressure of antibiotic use and the inability of vaccines to provide protection against many subtypes of APEC have promoted research into identifying alternative treatment approaches (Sgariglia et al., 2019; Mehat et al., 2021). The study of the differences in the responses of Silkie individuals, an important local chicken species used for both medicine and food, to APEC is helpful for formulating anti-APEC strategies. One of the purposes of this study was to screen APEC-resistant Silkie, which lays a foundation for subsequent studies of APEC-resistant genes.

MicroRNAs (miRNAs) are small noncoding RNAs that modulate gene expression at the posttranscriptional level either by inhibiting mRNA translation or by promoting mRNA degradation (Correia de Sousa et al., 2019). As essential regulators, many miRNAs are involved in regulating pathological and physiological processes after APEC infection (Jia et al., 2016; Sun et al., 2022a; Yin et al., 2022). Several studies have suggested that the miRNA expression profile may be a valuable biomarker for the diagnosis, prognosis, and prediction of response to therapy, as well as a powerful tool for disease prevention and treatment (Jia et al., 2016; Hrach and Mangone, 2019; Becker et al., 2021). However, alterations in miRNAs in susceptible and recovered Silkie chickens in response to APEC infection have not been reported. In this study, splenic miRNA expression among the APEC-susceptible, APEC-recovery, and control groups was investigated by small RNA sequencing. This study paves the way for screening APEC-resistant breeds and revealing the mechanism of APEC resistance and infection repair from a miRNA perspective.

## Materials and methods

### Ethical statement

The methods of this study were performed according to the Guidelines on the Administration of Experimental Animals established by the Committee of Science and Technology of Henan Province (China). The entire experimental protocol was approved by the Animal Care and Use Committee of Henan Agricultural University.

### Bacterial culture and count

The APEC O1 strain (CVCC1343), O2 strain (CVCC1344), and O78 strain (CVCC2941) were obtained from the China Institute of Veterinary Drugs Control. First, a freeze-dried powder of strain O1 was suspended in 400 μL of sterilized liquid Luria–Bertani (LB) medium. The suspension was plated on MacConkey agar and incubated overnight at 37°C. An isolated colony was selected and transferred into 4 mL of sterilized liquid LB medium and then incubated overnight at 37°C for 16 h with shaking. The O1, O2, and O78 strains were serially diluted and counted. The three *E. coli* strains were adjusted to a final concentration of 1×10^13^ colony-forming units (CFU)/mL. Then, the products were mixed at a 1:1:1 ratio. The mixed bacterial suspension was diluted to 1×10^6^ CFU/mL based on a 10-fold gradient.

### Chick infection in Experiment I

All 1-day-old, specific pathogen-free (SPF) Xichuan Silkie chicks were provided by the Poultry Genetic Resources Conservation Centre of Henan Agricultural University. In Experiment I, 45 chicks were randomly divided into nine groups with five chicks in each group. The chicks were acclimated for 8 days in a negative pressure isolator. On the pectoral muscles, the chicks in the different groups were inoculated with 0.5 mL of mixed bacterial suspensions of different concentrations (0 CFU/mL, 1×10^6^ CFU/mL, 1×10^7^ CFU/mL, 1×10^8^ CFU/mL, 1×10^9^ CFU/mL, 1×10^10^ CFU/mL, 1×10^11^ CFU/mL, 1×10^12^ CFU/mL, and 1×10^13^ CFU/mL) at 9 days of age. The experimental chicks were observed, and the data were recorded for 15 days. The data from Experiment I were used to calculate the median lethal dose of APEC.

### Anatomical symptoms and bacterial identification

During the 15-day observation period after APEC infection in Experiment I, the number of dead chicks was recorded, and the chicks were immediately dissected in the 24-h control room. Using an inoculation ring, the livers of these chicks were aseptically inoculated on MacConkey agar and eosin-methylene blue agar. This method was used to confirm that APEC infection was the cause of death.

### Determination of the median lethal dose

The Reed–Muench method was used to calculate the median lethal dose (LD_50_) (Stanic, 1963; Muthannan, 2016). In brief, we first calculated the “difference of logarithms”: difference of logarithms = [(mortality at dilution next above 50%) − 50%]/[(mortality at dilution next above 50%) − (mortality at dilution next below 50%)]. Then, we calculated the log_10_ 50% end point dilution:

log_10_ 50% endpoint dilution = log_10_ of dilution showing a mortality next above 50% − (difference of logarithms × logarithm of dilution factor) (Muthannan, 2016).

### Chick infection in Experiment II

A total of 360 one-day-old Silkie chicks were acclimated for 8 days in four negative pressure isolators (90/isolator). On the 9th day, 270 birds were intramuscularly inoculated with 0.5 mL of the mixed bacterial suspension containing APEC O1, O2, and O78 (LD_50_). An additional 90 control birds (Group C) were injected with 0.5 mL of sterilized saline. The morbidity and mortality of the chicks were recorded in detail for 15 days. Chicks that died within 15 days after APEC infection were classified as the susceptible group (Group S), and those that did not die were classified as the recovery group (Group R). Fifteen dead chicks in Group S were immediately dissected, and their splenic tissues were sampled. On the 15th day, 15 chicks in Group C and 18 in Group R were euthanized and necropsied. The remaining surviving chicks in Group C and Group R were separately raised to observe their later growth and incidence of disease for 4 months. The surviving chickens in Group R were used to breed the next generation, and the mechanism of anti-APEC was further studied. All spleen samples were stored in liquid nitrogen for further detection. Notably, 15 of the 18 chicks in Group R had inflammation in their internal organs, but 3 chicks in Group R had no inflammation.

### Library construction and small RNA sequencing

Total RNA was extracted from the splenic tissue in each sample with TRIzol reagent (Invitrogen, CA, USA) following the manufacturer’s procedure. Except for Group R, five RNA samples from the same group were equally pooled to form a single sequencing library. In Group R, three chicks without visceral inflammation were selected, and their RNA was mixed equally to form the R4 library. In the other 15 RNA samples, 5 were mixed equally to form the R1, R2, and R3 libraries. Approximately 1 µg of total RNA was used to prepare a small RNA library according to the protocol of the Small RNA Library Prep Kit (KAITAI-BIO, China). Ten small RNA libraries (C1, C2, C3, S1, S2, S3, R1, R2, R3, and R4) were constructed. Then, double-end sequencing was performed on an Illumina HiSeq Xten (Hangzhou, China) following the vendor’s recommended protocol. KAITAI-BIO Company (China) provided assistance with the small RNA sequencing. Because the raw data had adapter reads, low-quality reads, and fragment lengths <18 bp or >36 bp, the raw data were filtered with fastp software to obtain clean data. The clean data were aligned to the reference genome (*Gallus gallus* bGal1.mat.broiler. GRCg7b) to obtain the mapped reads. We used Bowtie software to annotate the RNA families and miRdeep2 software (https://www.mdcberlin.de/8551903/en/) to predict new miRNAs in the mapped reads. In this process, novel miRNAs were identified by binding to homologous miRNA sequences of related species. The expression level of miRNA in each sample was normalized using reads per million (RPM) values. The miRNA expression profiling data in this study have been deposited in the Genome Sequence Archive in the National Genomics Data Center, China National Center for Bioinformation/Beijing Institute of Genomics, Chinese Academy of Sciences (GSA: CRA009953), which are publicly accessible at https://ngdc.cncb.ac.cn/gsa.

### Sample clustering analysis

Pearson’s correlation coefficient (*r*) is an indicator of biological replicate correlation. The closer the *r* value is to 1, the stronger the correlation between two repeated samples is. Pearson’s correlation coefficient of each sample in pairs was calculated to construct a clustering heatmap. We also performed principal component analysis (PCA) based on miRNA expression. If the first principal component is projected on the first coordinate and the second principal component is projected on the second coordinate, the PCA method can be used to visualize the clustering of samples.

### Analysis of differentially expressed miRNAs

Based on the expression levels of the normalized miRNAs, differentially expressed (DE) miRNAs were identified using the edgeR algorithm R package. A false discovery rate (FDR) <0.05 and a |log_2_ (fold change)| >0.58 were used to screen for known DEmiRNAs. Venn plots, fold change plots, volcano plots, and heatmaps were drawn to visualize the known DEmiRNAs among the samples.

### Functional analysis of the DEmiRNAs

miRNAs perform regulatory functions by negatively regulating the expression of target genes through binding to the 3’ UTR of the target gene. Based on the complementary relationship between the miRNA seed region and the 3’ UTR of the target gene, combined with free energy, the target gene of the miRNA can be predicted. To improve the prediction accuracy, the genes identified by the intersection of the miRanda and TargetScan predictions were used as miRNA-targeted genes. The target genes of the DEmiRNAs were subjected to Gene Ontology (GO) and Kyoto Encyclopedia of Genes and Genomes (KEGG) enrichment analyses using ClusterProfiler (http://bioconductor.org/packages/release/bioc/html/clusterProfiler.html) in the R package. Cytoscape (http://www.cytoscape.org/) was used to construct a visual network to analyze the regulatory relationships between the DEmiRNAs and their potential target genes.

## Results

### Morbidity and mortality in Experiment I

Six hours after the injection of the mixed bacterial suspension (O1, O2, and O78), the chicks in the different dose groups exhibited depression, drowsiness, closed eyes, an unwillingness to walk, ruffled feathers, drooping wings, shivering, diarrhea, and no appetite. The chicks in the high-dose group began to die 16 h after challenge, and the peak time of death was in the first 3 days. Only a small number of chicks died in the following 7 days. No chicks died after day 10. There was no morbidity or death in the control group (**Table 1**).

**Table 1:** The mortality of chicks injected with different doses of the *E. coli* mixture within 15 days.

Day Dose	D9	D10	D11	D12	D13	D14	D15	D16	D17	D18	D19	D20	D21	D22	D23	D24
0.5 mL×10^13^			1	1		1				1						
0.5 mL×10^12^		1			1					1						
0.5 mL×10^11^		1		1												
0.5 mL×10^10^				1												
0.5 mL×10^9^		1														
0.5 mL×10^8^																
0.5 mL×10^7^																
0.5 mL×10^6^			1								1					
0																

### Median lethal dose

Based on the data in **Table 2**, the median lethal dose was determined to be 1.45×10^11^ CFU.

**Table 2:** The mortality after APEC infection.

Bacterial dilution	Dose	Observations		Cumulative results			
		Number of deaths	Number of survivors	Number of deaths	Number of survivors	Total	Mortality (%)
10^0^	0.5 mL×10^13^	4	1	13	1	14	92.86
10^−1^	0.5 mL×10^12^	3	2	9	3	12	75.00
10^−2^	0.5 mL×10^11^	2	3	6	6	12	50.00
10^−3^	0.5 mL×10^10^	1	4	4	10	14	28.57
10^−4^	0.5 mL×10^9^	1	4	3	14	17	17.65
10^−5^	0.5 mL×10^8^	0	5	2	19	21	9.52
10^−6^	0.5 mL×10^7^	0	5	2	24	26	7.69
10^−7^	0.5 mL×10^6^	2	3	2	27	29	6.90

### Anatomical symptoms and bacterial identification of dead chickens

**Figure 1:**
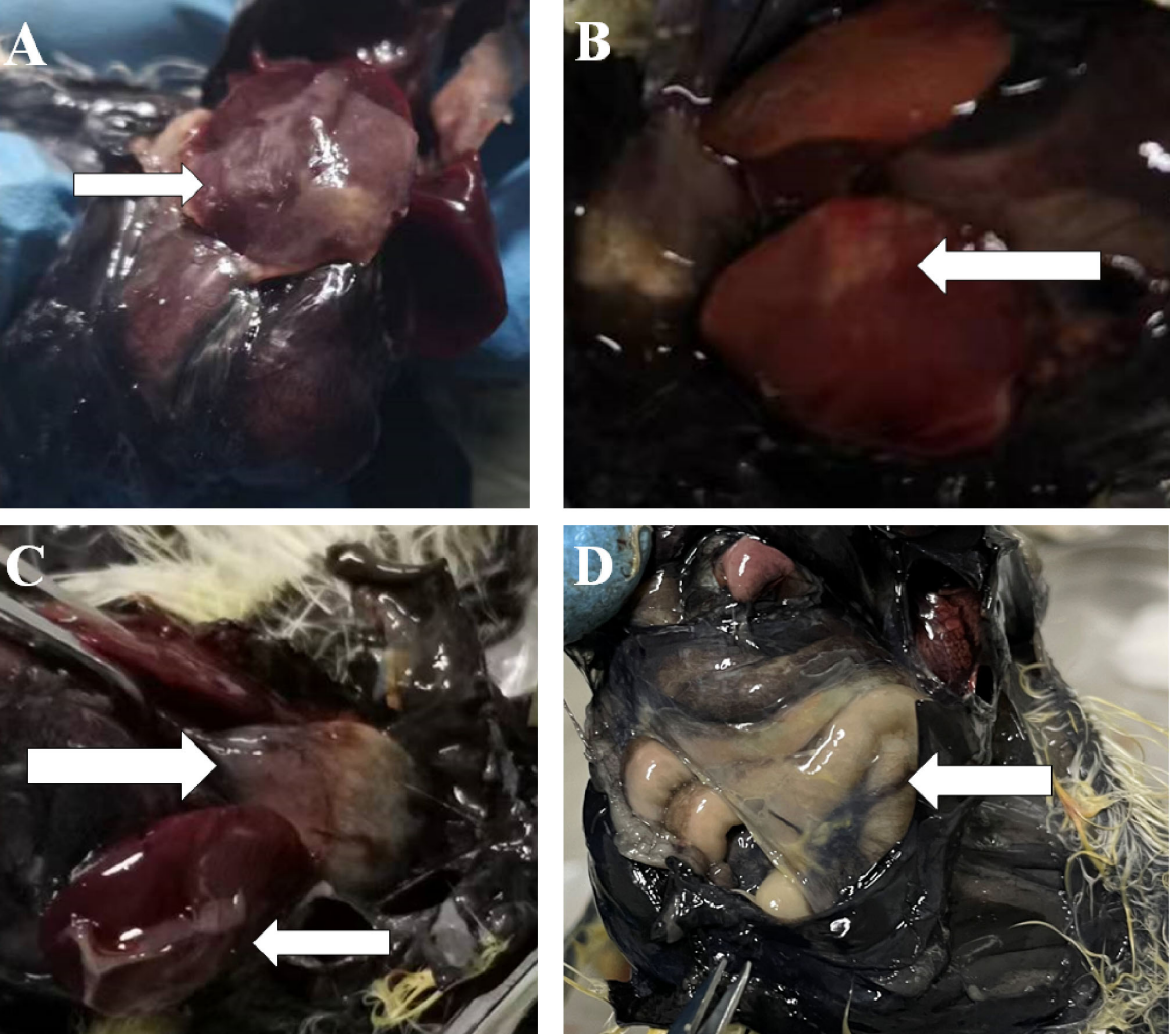
Part of the histogram. The white arrows indicate the site of the lesion. **(A, B)** Morphology of liver lesions. **(C)** Morphology of liver and heart lesions. **(D)** Morphology of the air sac lesions.

Chickens infected with APEC in Experiment I were dissected immediately after death. For chickens that died within 16 h, there were small bleeding spots on the intestinal serosa, endocardium, and epicardium. The intestine was inflatable and contained a large amount of mucus. For chickens 24 h after death, the liver membrane presented as a frosted fibrinous capsule (**Figures 1A, C**), and the liver was enlarged and necrotic with white spots or plaques (**Figure 1B**). The pericardium was thickened and opaque, the pericardial fluid was cloudy, and there was a large yellow fatty deposit (**Figure 1C**). The air sacs were cloudy or adhered to cheese-like substances (**Figure 1D**).

The livers of the dead chickens were inoculated on MacConkey agar. The bacteria grew well and produced pink colonies (**Figure S1**). Single colonies were sampled and stained. Microscopic examination revealed Gram-negative bacteria. Similarly, the livers of dead chickens were inoculated on eosin-methylene blue agar. The bacteria grew well, and the colonies were purple−black (**Figure S1**), with surfaces having a metallic luster, which was in accordance with the characteristics of *E. coli* culture. Therefore, it was concluded that the chicks died of *E. coli* infection.

### Morbidity and mortality in Experiment II and late survival

After 24 h, 83 of the 270 chicks died (**Table 3**). Most deaths occurred on the second and third days after injection. By the 8th day, the chicks were no longer dying. Those that survived 15 days after infection were kept for 4 months, during which no vaccine was injected, and no deaths occurred.

**Table 3:** The number of dead chicks per day after APEC infection with LD_50_.

Day	D9	D10	D11	D12	D13	D14	D15	D16	D17	D18	D19	D20	D21	D22	D23	D24
Number of deaths	0	83	24	3	7	8	1	0	0	0	0	0	0	0	0	0
Total of deaths	126															
Mortality	126/270 = 46.7%															

### An overview of the detected miRNAs and the clustering of samples

**Figure 2:**
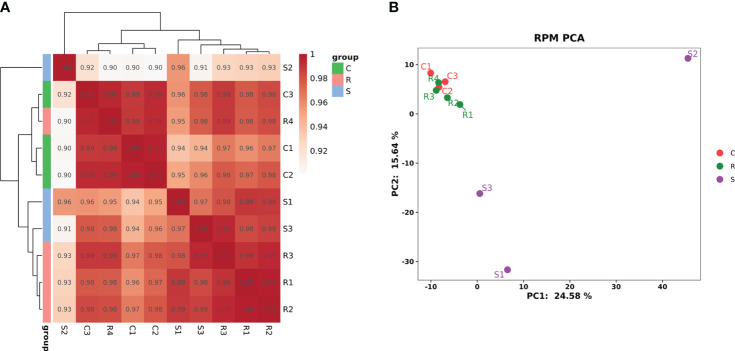
The cluster diagram of the sample. **(A)** Clustering of samples based on calculated Pearson’s correlation coefficients. **(B)** Clustering of samples based on PCA.

The RNA family distribution of each sample obtained by small RNA sequencing analysis is shown in **Figure S2**. A total of 1,144 miRNAs were detected in the three groups; 759 were known miRNAs, and 385 were novel miRNAs (**Figure S3**). Clustering analysis of Pearson’s correlation coefficient showed that R4 clustered with the normal controls; R1, R2, and R3 clustered; and S1 clustered closer to S3 but farther from S2 (**Figure 2A**). PCA showed that the four Group R replicates were closer to the three Group C replicates but were farther from the three Group S replicates. Moreover, S2 was farther from S1 and S3 (**Figure 2B**).

### Analysis of the DEmiRNAs in response to APEC infection

**Figure 3:**
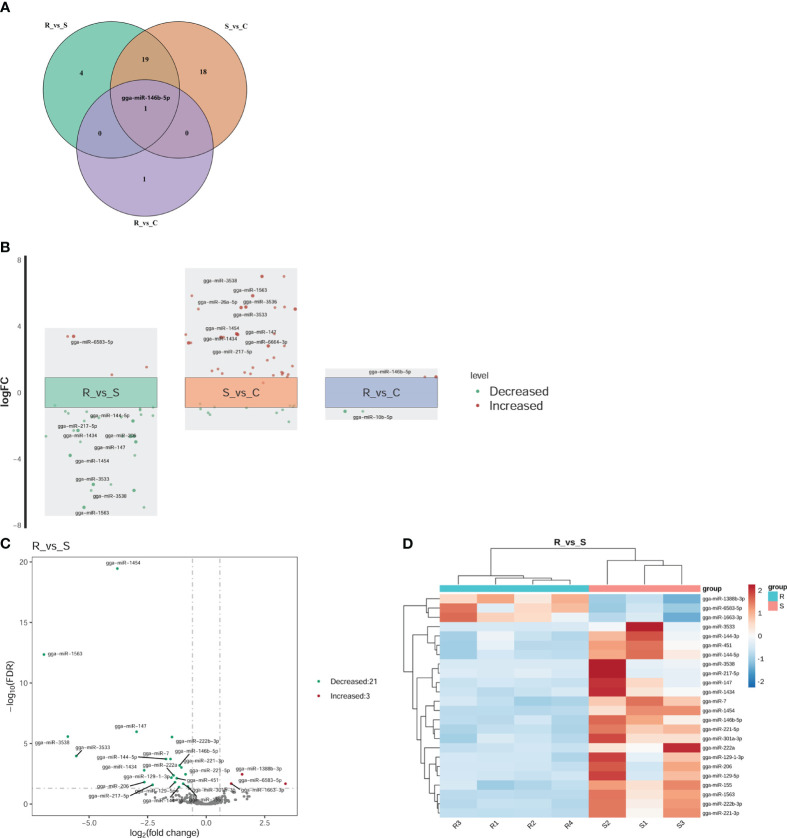
Differential expression analysis of miRNAs. **(A)** Venn diagram of the DEmiRNAs within the three comparisons of R vs. S, R vs. C, and S vs. (C) gga-miR-146b-5p was the only shared miRNA across all three comparisons. **(B)** Fold change (FC) plot of the DEmiRNAs. The vertical coordinate is the logarithm of the FC of miRNA expression between two groups. The nearest 0 are miRNAs with no significant differences, indicated by boxes. Above the box are miRNAs with 1.5-fold upregulation, indicated by red dots; below the box are miRNAs with 1.5-fold downregulation, indicated by green dots. The top 10 miRNAs according to the fold change are shown in the diagram. **(C)** Volcano plot of all DEmiRNAs in Group R and Group S. The horizontal coordinate represents the logarithm of the fold change, and the zone between the two dotted lines represents the miRNAs with no significant difference. The longitudinal coordinate is the logarithm of the false discovery rate (FDR), representing the significance level. An FDR < 0.05 was considered to indicate a significant difference. **(D)** Cluster heatmap of the 24 DEmiRNAs in the Group R vs. Group S comparison. Dark red indicates miRNAs in the sample expressed at high levels, and blue indicates miRNAs in the sample expressed at low levels.

Twenty-four DEmiRNAs were identified for the R vs. S comparison, 3 of which were upregulated and 21 of which were downregulated (**Figures 3A, D**). Among the 24 DEmiRNAs, 8 had high expression levels (RPM > 100): gga-miR-222b-3p, gga-miR-146b-5p, gga-miR-221-3p, gga-miR-221-5p, gga-miR-222a, gga-miR-451, gga-miR-155, and gga-miR-144-3p. Thirty-eight DEmiRNAs were identified for the S vs. C comparison, 26 of which were upregulated and 12 of which were downregulated (**Figure 3A**). Two DEmiRNAs were identified for the R vs. C comparison, one of which was gga-miRNA-146b-5p (upregulated), and the other was miRNA-10b-5p (downregulated) (**Figures 3A, B**). The expression levels of these genes were greater than 100 RPM. gga-miRNA-146b-5p was the only co-DE gene among the DEmiRNAs according to pairwise comparison (**Figure 3A**). In the comparison of R vs. S and S vs. C, the top two genes based on fold changes were gga-miRNA-1563 and gga-miRNA-3538 (**Figure 3B**). The expression levels of miRNAs in Group R were similar to those in Group C, except for gga-miRNA-146b-5p and gga-miRNA-10b-5p (**Figure 3B**). These two miRNAs may play regulatory roles in recovery after APEC infection. The results above suggested that the regulatory effect of miRNAs was widespread in response to APEC infection. In all three groups, we focused on the changes based on the R vs. S comparison because both groups experienced APEC infection. Therefore, the fold changes for all DEmiRNAs and the FDRs based on the R vs. S comparison are shown in **Figure 3C**. Clustering analysis of 24 DEmiRNAs revealed that the four replicates in Group R were different from the three replicates in Group S (**Figure 3D**). Moreover, the three replicates in Group S had their own different characteristics in terms of the expression of miRNAs (**Figure 3D**). This suggested that after APEC infection, susceptible chickens might develop lesions of varying degrees at different sites throughout the body and that the DEmiRNAs respond to these differential lesions.

### GO analysis and KEGG analysis

**Figure 4:**
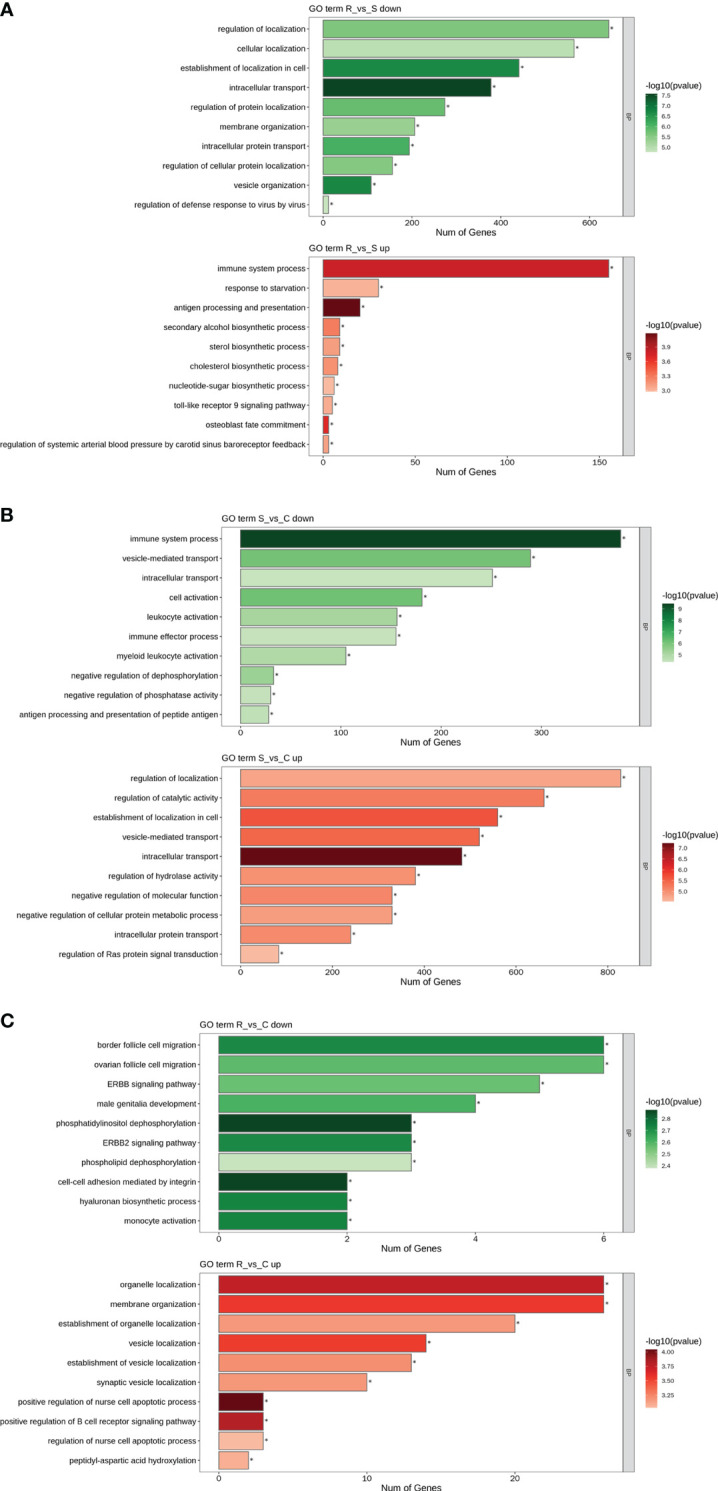
GO term analysis of DEmiRNAs. Only the biological process (BP) in the GO analysis is shown in all the figures. Green indicates GO analysis of target genes of downregulated miRNAs. Red indicates GO analysis of target genes of upregulated miRNAs. The length of the column represents the number of genes. The shade of the color indicates the level of significance. **(A)** R vs. S. **(B)** S vs. (C) **(C)** R vs. C.

**Figure 5:**
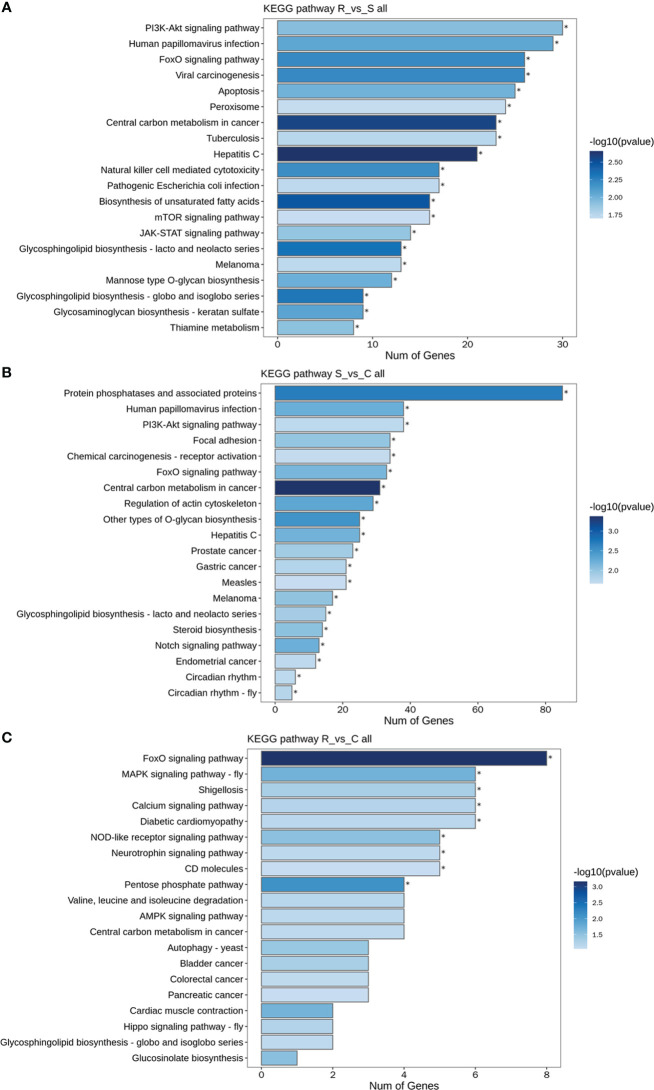
KEGG pathways of DEmiRNAs. **(A)** All DEmiRNAs for the R vs. S comparison. **(B)** All DEmiRNAs for the S vs. C comparison. **(C)** All DEmiRNAs for the R vs. C comparison.

GO and KEGG analyses were performed to investigate the functions of the DEmiRNAs. Compared with those in Group S, the downregulated miRNAs in Group R were involved mainly in transport- and localization-related biological process-related GO terms (**Figure 4A**). The upregulated miRNAs identified in the R vs. S comparison were enriched in immune-related GO terms, including immune system process, antigen processing and presentation, and the Toll-like receptor 9 signaling pathway (**Figure 4A**). Additionally, the significantly enriched KEGG pathways based on the Group R vs. Group S comparison were the PI3K-Akt signaling pathway, human papillomavirus infection, the FoxO signaling pathway, viral carcinogenesis, apoptosis, peroxisomes, central carbon metabolism in cancer, tuberculosis, hepatitis C, natural killer cell-mediated cytotoxicity, and pathogenic *E. coli* infection (**Figure 5A**). Similarly, many GO terms related to the DEmiRNAs identified in the S vs. C comparison were also involved in immunomodulation, including immune system processes, leukocyte activation, immune effector processes, myeloid leukocyte activation, antigen processing and presentation of peptide antigens, and regulation of Ras protein signal transduction (**Figure 4B**). Additionally, the largest number of genes that were targeted by the DEmiRNAs based on the S vs. C comparison were enriched in the pathway “central carbon metabolism in cancer” (**Figure 5B**). When Group R was compared with Group C, many GO terms associated with downregulated miRNAs were related to “phosphatidylinositol dephosphorylation” and “cell−cell adhesion mediated by integrin”, and the GO terms associated with upregulated miRNAs were mainly related to “positive regulation of nurse cell apoptotic process” and “positive regulation of B-cell receptor signaling pathway” (**Figure 4C**). Additionally, the KEGG pathway enriched with the most genes and with the most significance was the FoxO signaling pathway (**Figure 5C**).

### Construction of the miRNA−mRNA network

**Figure 6:**
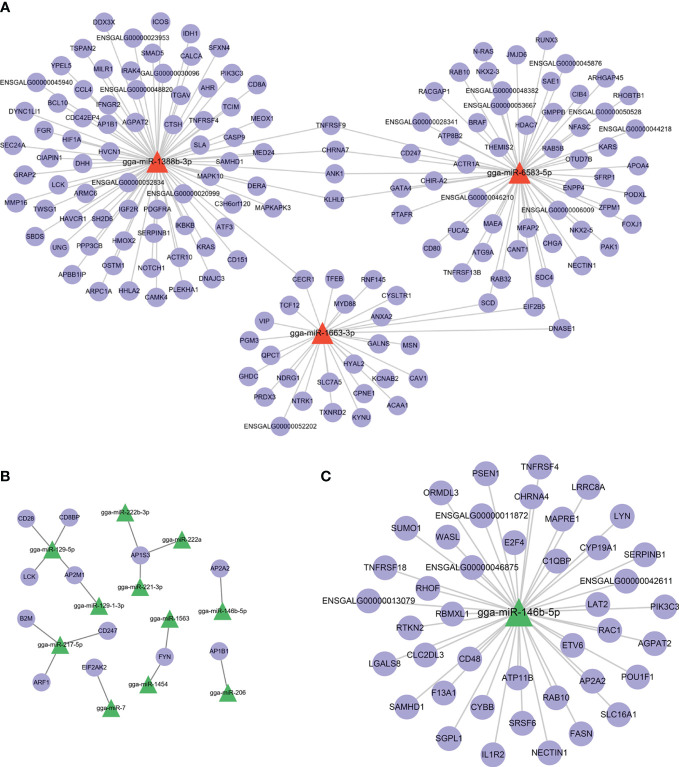
miRNA−target gene network. **(A)** shows the network of upregulated miRNAs (R vs. S) and their target genes. **(B)** shows the network of downregulated miRNAs (R vs. S) and their target genes. **(C)** The network of gga-miRNA-146b-5p and its target genes related to immunity.

Because both Group R and Group S were infected with APEC, the DEmiRNAs between them may have potential biological functions in preventing APEC infection or repairing post-APEC infection. Upregulated miRNAs (R vs. S) with target genes associated with “immune system process” (GO:0002376) and downregulated miRNAs (R vs. S) with target genes associated with “regulation of defense response to virus by virus” (GO:0050690) were selected, and miRNA−target gene networks were constructed (**Figures 6A, B**). Moreover, miRNA−target gene networks associated with “antigen processing and presentation” (GO:0019882) and “Toll-like receptor 9 signaling pathway” (GO:0034162) were also constructed (**Figure S4**). Based on the role of the networks and the abundance of miRNAs, these DEmiRNAs (gga-miR-1663-3p, gga-miR-222b-3p, gga-miR-146b-5p, gga-miR-221-3p, gga-miR-222a, and gga-miR-7) play important roles in the response to APEC infection. As the only co-DEmiRNA identified from a pairwise comparison of the three groups, gga-miRNA-146b-5p was chosen for the construction of a separate network with its target genes (**Figure 6C**). Many immune-related genes, such as IL1R2, CD48, and TNFRSR4, were found to be potential targets of gga-miRNA-146b-5p (**Figure 6C**).

## Discussion

There are no desirable means of preventing or controlling APEC infection, one of the most common bacterial infections in the world. In poultry farming, developing disease-resistant breeds is a feasible strategy in an effort to reduce the use of antibiotics and vaccines. In this study, we first determined the median lethal dose of a mixture of APEC serotypes (O1, O2, and O78) in Silkie chickens. In several previous studies, chickens were infected with isolated strains of APEC O1 or O78 at a dose of 10^8^ CFU (Sandford et al., 2011; Jia et al., 2016; Yin et al., 2022; Shi et al., 2023). Globally, O1, O2, and O78 are the predominant O-antigen serotypes of APEC (Li et al., 2018). Therefore, the use of a mixture of three strains was more comprehensive for screening APEC-resistant breeds. This study is the first to determine the median lethal dose of APEC in Silkie (1.45×10^11^ CFU, intrathoracic injection, 9 days old), providing a dose basis for subsequent study.

Previous studies have reported differences in gene expression and miRNA expression between APEC-resistant and APEC-susceptible chickens. The distinction between the resistant and the susceptible chickens in their experiment was based on weak or severe symptoms 72 h after disease onset (Sandford et al., 2011, 2012; Shi et al., 2023). In this study, APEC-susceptible or APEC-resistant birds were selected according to death or nondeath within 15 days postinfection. The chickens that did not die after 15 days of infection reached the recovery period. We reared chickens that survived 15 days after APEC infection for 4 months. The chickens were not vaccinated against any diseases during the 4-month period, but no deaths occurred in the flock. The chickens are used to breed the next generation for future APEC-resistant breeding studies.

As the largest immune organ in poultry, the spleen plays a very important role in the response to disease (Sandford et al., 2011; Jia et al., 2016). The regulatory role of splenic miRNAs in response to APEC infection is of interest. Significantly, DEmiRNAs were detected between the susceptible and recovery groups. Although the sampling times were not consistent between the susceptible and recovery groups, the changes in miRNA expression in the recovery group were also different from those in the control group (for which the sampling times were consistent), suggesting that the DEmiRNAs (not only from Group R versus Group S but also from Group R versus Group C) may play an important role in resistance and recovery after infection. Chickens in Group S were also sampled at different times, but miRNAs from chickens with S1 and S3 repeats were clustered together, suggesting that sampling time can be disregarded over a shorter period and that physiological state consistency should be considered more. The DEmiRNAs were shown to be involved in many immune-related biological processes, including immune system process, antigen processing and presentation, the Toll-like receptor 9 signaling pathway, and regulation of defense response to virus by virus. Consistent with reports by Yin et al., they also found that the GO term “immune system processes” was significantly enriched in the bone marrow of chickens infected with APEC when functional annotation analysis of target genes of DEmiRNAs was performed (Yin et al., 2022). In this study, “immune system process” was significantly upregulated in Group R compared with Group S or Group C compared with Group S. Therefore, “immune system process” may play an essential role in resistance to APEC infection. The enriched pathways of the DEGs included natural killer cell-mediated cytotoxicity, pathogenic *E. coli* infection, and CD molecules, which are directly related to immunity. In addition, the MAPK and Notch signaling pathways are triggered in response to APEC infection, which was consistent with the findings of Yin et al. (2022). In addition, the FoxO signaling pathway appeared in the overlap of pairwise comparisons and should be given additional attention. The DE genes were also shown to be involved in the FoxO signaling pathway in tests of *Salmonella enteritidis* infection in poultry, and this pathway was strongly activated in susceptible poultry (Li et al., 2018). In our study, the activation of the FoxO signaling pathway under different conditions might be associated with the APEC infection response and postinfection repair.

We identified gga-miR-1663-3p, gga-miR-222b-3p, gga-miR-146b-5p, gga-miR-221-3p, gga-miR-222a, and gga-miR-7 as key miRNAs involved in survival after APEC infection. Quero et al. reported that miR-221-3p could drive the shift of M2 macrophages from an anti-inflammatory phenotype to a proinflammatory phenotype (Quero et al., 2020). Shen et al. reported that miR-221-3p could enhance smoking-induced inflammation in chronic obstructive pulmonary disease (Shen et al., 2021). In this study, the expression of gga-miR-221-3p was greater in the susceptible group than in the resistant group, and in combination with the above findings in humans, we speculated that gga-miR-221-3p has a proinflammatory effect on APEC infection. miR-222a, another highly expressed miRNA in the susceptible group, can act as an antiviral factor against duck hepatitis A virus 1 (Sui et al., 2022). However, few other miRNAs, such as gga-miR-1663-3p, gga-miR-222b-3p, and gga-miR-7, have been reported. Therefore, further study of its function was performed to determine its role in APEC infection.

In the present study, gga-miR-146b-5p was found to be a coexpressed miRNA that was continuously and differentially upregulated from the control to the recovery to susceptible groups. Jia et al. also reported that gga-miR-146b-5p was not only DE but also highly expressed in the splenic response to APEC infection (Jia et al., 2016). However, most studies on miR-146b-5p have focused mainly on humans. It was reported that miR-146b-5p is involved in lipopolysaccharide (LPS)-evoked injury and the inflammatory response in human small airway epithelial cells and that miR-146b-5p inhibits the inflammatory response and apoptosis via JAK1/STAT1 signaling in LPS-induced glomerular cells (Gao and Zhang, 2021; Zhang et al., 2021). In addition, miR-146b-5p overexpression alleviates neonatal hypoxic ischemic encephalopathy-induced neuronal injury by inhibiting the IRAK1/TRAF6/TAK1/NF-κB pathway (Yang and Zhao, 2020). In the present study, gga-miR-146b-5p was predicted to target many immune-related genes, but the specific role of gga-miR-146b-5p in immune and inflammatory responses to APEC needs further investigation.

## Conclusion

In general, Silkie resistance to APEC infection varies among individuals. Selecting APEC-resistant breeds to address APEC challenges may be an effective strategy. According to a comparison of susceptible, recovering, and control Silkies, certain miRNAs were identified, and “immune system process” and “FoxO signaling pathway” were shown to play important roles in the APEC infection response and postinfection repair. These findings lay the foundation for APEC-resistant breeding in poultry and contribute to our understanding of susceptibility and repair to APEC infection through miRNA-induced systems.

## Data availability statement

The datasets presented in this study can be found in online repositories. The names of the repository/repositories and accession number(s) can be found below: https://ngdc.cncb.ac.cn/gsa, GSA: CRA009953.

## Ethics statement

The animal studies were approved by the Animal Care and Use Committee/Henan Agricultural University. The studies were conducted in accordance with the local legislation and institutional requirements. Written informed consent was obtained from the owners for the participation of their animals in this study.

## Author contributions

WQL: Conceptualization, Resources, Software, Supervision, Writing – original draft, Writing – review & editing. WLL: Conceptualization, Funding acquisition, Supervision, Writing – original draft, Writing – review & editing. PW: Data curation, Formal analysis, Methodology, Software, Writing – review & editing. WJ: Data curation, Investigation, Methodology, Software, Writing – review & editing. LY: Data curation, Formal analysis, Methodology, Validation, Writing – review & editing. BW: Data curation, Formal analysis, Methodology, Validation, Writing – review & editing. SL: Investigation, Methodology, Validation, Writing – original draft. XK: Conceptualization, Resources, Supervision, Writing – original draft, Writing – review & editing.

## Supplementary material

Supplementary Figure 1 Morphology of bacteria on two different media. **(A)** MacConkey agar; **(B)** Eosin-methylene blue agar.

Supplementary Figure 2 Pie plot of the small RNA distribution.

Supplementary Figure 3 Venn diagram of the known and novel miRNAs in the three groups. **(A)** indicates the known miRNAs. **(B)** indicates the novel miRNAs.

Supplementary Figure 4 Supplementary upregulated miRNA−target gene network (R vs. S). **(A, B)** indicate the network associated with “antigen processing and presentation”. **(C)** indicates the network associated with the “Toll-like receptor 9 signaling pathway”.
