# Supplementary figures and images for: Differential responses to avian pathogenic E. coli and the regulatory role of splenic miRNAs in APEC infection in Silkie chickens
Source: Front Cell Infect Microbiol. 2024 Mar 12;14:1358216. doi: 10.3389/fcimb.2024.1358216 (PMC10963617; doi:10.3389/fcimb.2024.1358216)

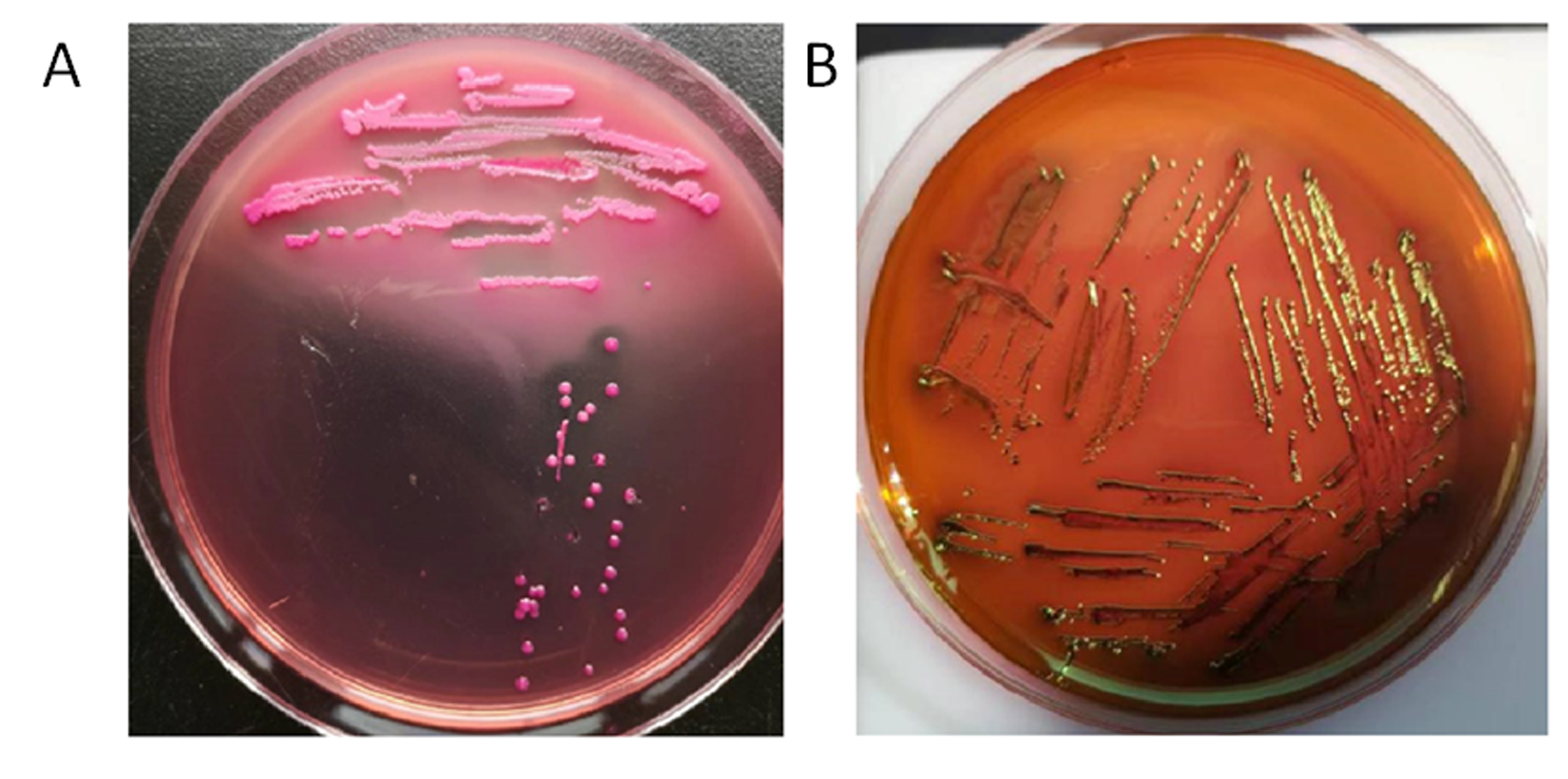

Supplement: Supplementary Figure 1 — Morphology of bacteria on two different media. (A) MacConkey agar; (B) Eosin-methylene blue agar. [file Image_1.tif]

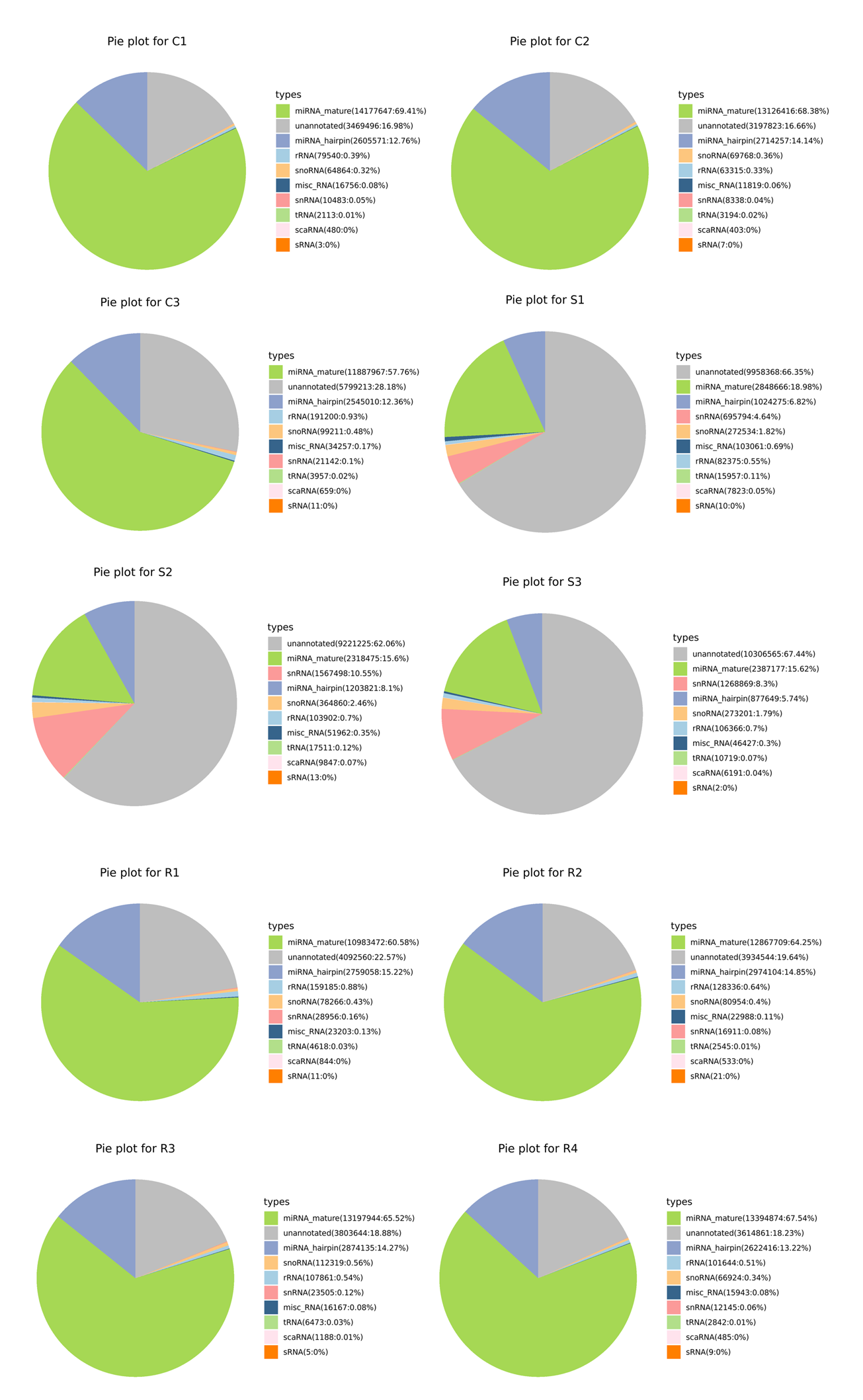

Supplement: Supplementary Figure 2 — Pie plot of the small RNA distribution. [file Image_2.tif]

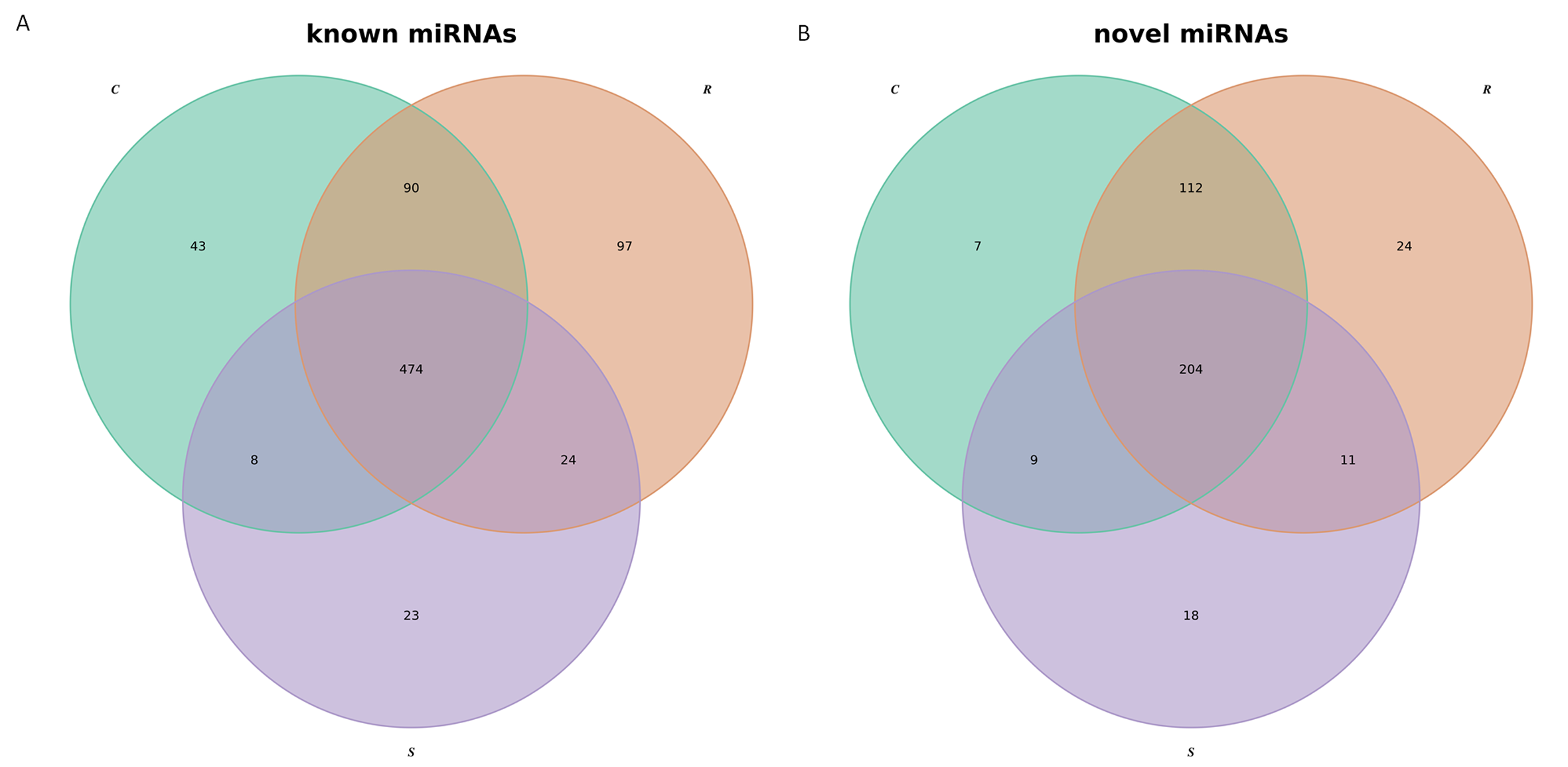

Supplement: Supplementary Figure 3 — Venn diagram of the known and novel miRNAs in the three groups. (A) indicates the known miRNAs. (B) indicates the novel miRNAs. [file Image_3.tif]

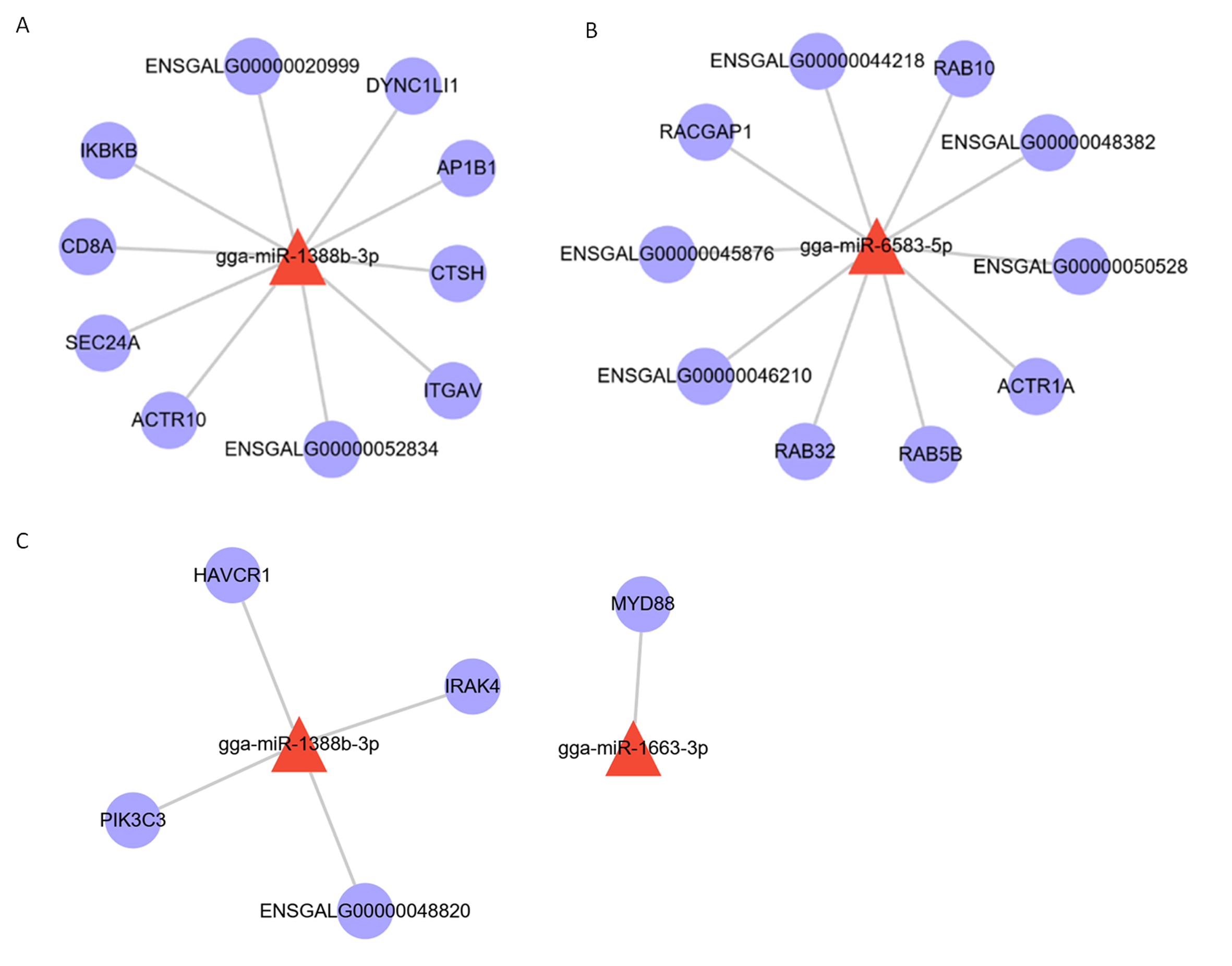

Supplement: Supplementary Figure 4 — Supplementary upregulated miRNA−target gene network (R vs. S). (A, B) indicate the network associated with “antigen processing and presentation”. (C) indicates the network associated with the “Toll-like receptor 9 signaling pathway”. [file Image_4.tif]
